# Measuring the intensity of emotions

**DOI:** 10.3389/fpsyg.2024.1437843

**Published:** 2024-09-02

**Authors:** Rainer Reisenzein, Martin Junge

**Affiliations:** Institute of Psychology, University of Greifswald, Greifswald, Germany

**Keywords:** emotion intensity, difference measurement, difference scaling, testing measurement axioms, indirect scaling methods, rating scales, emotion measurement

## Abstract

We describe a theoretical framework for the measurement of the intensity of emotional experiences and summarize findings of a series of studies that implemented this framework. Our approach is based on a realist view of quantities and combines the modern psychometric (i.e., latent-variable) view of measurement with a deductive order of inquiry for testing measurement axioms. At the core of the method are nonmetric probabilistic difference scaling methods, a class of indirect scaling methods based on ordinal judgments of intensity differences. Originally developed to scale sensations and preferences, these scaling methods are also well-suited for measuring emotion intensity, particularly in basic research. They are easy to perform and provide scale values of emotion intensity that are much more precise than the typically used, quality-intensity emotion rating scales. Furthermore, the scale values appear to fulfill central measurement-theoretical axioms necessary for interval-level measurement. Because of these properties, difference scaling methods allow precise tests of emotion theories on the individual subject level.

## 1 Introduction

Linguistic and phenomenological evidence indicates that emotions—by which we mean emotional experiences—differ from each other not only in type or quality, but also in intensity. For example, we say not only that someone, including ourselves, is happy, sad, or surprised; we often qualify these emotion ascriptions with intensity modifiers such as “a little,” “moderately,” or “extremely”: Karl is a little happy, Maria is moderately sad, we feel extremely surprised. These linguistic practices are supported by introspection, which confirms that different episodes of joy, sadness etc. can differ greatly in intensity, and that even during an emotion episode of constant quality, the intensity of the feeling can wax and wane. Generalizing these observations, one may say that linguistic and phenomenological evidence indicates that each emotion type can occur in different degrees or gradations, ranging from just noticeable to extremely intense.

This generalization suggests the hypothesis that emotions are *quantities*, that is, continuous magnitudes with an additive structure (see Michell, 1999 and Section 5). If so, theories of emotion should preferably be quantitative theories, that is theories in which magnitudes are connected by numerical functions (Carnap, 1966). However, stringent tests of these theories require measuring the intensity of emotions on a metric (interval or ratio) scale level. If emotions are indeed quantities, this should be possible in principle, i.e., provided suitable measurement methods can be devised. Indirect support for these assumptions is provided by the observation (Reisenzein, 2012) that, in being a group of related phenomenal qualities graded in intensity, emotions are similar to sensations (e.g., of tone, touch, or temperature). Sensations, however, are generally regarded as quantities, and it is also widely believed that their intensity can be measured on a metric scale level (e.g., Stevens, 1975; Anderson, 1981; Schneider, 1982; Marks and Gescheider, 2002; Kingdom and Prins, 2010).

As a matter of fact, the assumption that emotional feelings, like sensations, are quantities whose intensity can therefore in principle be measured on a metric scale, has been made since the beginnings of academic psychology in the 19th century (e.g., Fechner, 1871; Külpe, 1893; Wundt, 1896; Titchener, 1902); and it continues to be held, at least implicitly, by probably most of today’s emotion researchers. What is controversial, however, is how a precise, metric measurement of emotion intensity can be achieved.

This issue is particularly contentious regarding the most frequently used method for assessing the intensity of emotional feelings, the direct scaling of emotion intensity on quality-intensity rating scales (e.g., “How happy are you right now on a scale from 0 = not at all to 10 = extremely?”). The fact that most emotion researchers analyze these data with statistical methods that presuppose a metric scale level (e.g., linear regression), suggests that they believe that emotion intensity ratings are at least approximately metric. This view has been defended, for rating scales more generally, by several authors, most elaboratedly by Anderson (1981, 1982). In contrast, critics of rating scales insist, with equal tenacity, that rating scales are only ordinal and their analysis with metric statistical methods is therefore problematic, if not outright illegitimate (for a recent version of this critique see Liddell and Kruschke, 2018). Attempts to test the assumption that emotion rating scales—or, for that matter, other methods of measuring the intensity of emotional experiences—yield metric scales, are however exceedingly rare.

In view of the contested scale level of rating scales, as well as the many other criticisms raised against them (see Section 2.2.1), we have during the past years explored alternative methods of measuring emotion intensity that avoid the problems of rating scales and yield a metric scale level, or a least approach the metric level more closely than rating scales do (Junge and Reisenzein, 2013, 2015, 2016; Reisenzein and Franikowski, 2019; Reisenzein and Junge, 2024). As part of this research project, we also tested the metricity of emotion intensity ratings (Junge and Reisenzein, 2016). We found a suitable class of methods in probabilistic nonmetric difference scaling methods, a class of indirect scaling methods originally developed in psychophysics and preference measurement. Its main variants are *Ordinal Difference Scaling* (Agresti, 1992; Boschman, 2001; see also Tutz, 1986) and *Maximum Likelihood Difference Scaling* (Maloney and Yang, 2003; Knoblauch and Maloney, 2008). These methods have been successfully applied in the sensory and perceptual domain (e.g., Boschman, 2001; Maloney and Knoblauch, 2020), but prior to our studies, they were not used for the scaling of emotion intensity.

In this article, we summarize our research and elaborate and justify the theoretical approach to emotion intensity measurement that it exemplifies. Briefly, our approach is founded on a realist view of measurement (see, e.g., Michell, 1999, 2005; Tal, 2020) and combines the modern psychometric (i.e., latent-variable) approach to measurement (see, e.g., Borsboom, 2005) with a deductive order of inquiry of testing measurement axioms (Westermann, 1983, 1985). Although the components of this approach to mental measurement are not new, certain elaborations of these components are (see in particular Section 5.3), as is the application of the proposed method to the measurement of emotion intensity. The main part of the article describes our approach to emotion measurement and the findings obtained with it. This part is preceded by a brief review of scaling methods that have been used to measure the intensity of emotional experiences.

Before proceeding, it is important to emphasize that the proposed indirect scaling method is not intended to replace emotion ratings or other direct intensity scaling methods in all situations. As discussed in Section 5, difference scaling is not suitable for all measurement contexts, and is more costly than direct scaling methods. Nevertheless, we believe that in research contexts where difference scaling can be used, its additional costs are often a worthwhile trade-off for obtaining more precise, less biased, and closely metric measurements.

## 2 Methods for measuring the intensity of emotional experiences

### 2.1 The object of measurement: emotional states

When speaking of *emotions* in this article, we mean *occurrent emotional states*, such as an episode of joy, sadness, fear, or relief. Emotional states are temporary mental states of typically short duration, that are at least normally conscious, and are typically evoked by perceptions or thoughts of motivationally relevant objects or events. As conscious mental states, emotions are characterized by a more or less emotion-specific experiential quality that occurs with a particular intensity, and they are usually experienced as being directed at the evoking objects (e.g., Karl is happy about the arrival of a friend). Emotional states are what emotion psychologists are first and foremost interested in, and what theories of emotion are primarily about (see, e.g., Reisenzein, 2015). Our focus on emotional states means that we ignore here the measurement of *emotional dispositions*, i.e., tendencies or readinesses to have particular emotional states in suitable situations (see Reisenzein et al., 2020).

Although a definitive list of the mental states that count as emotions does not exist (see Reisenzein, 2012, for a discussion), there is broad agreement among emotion researchers, as well as lay people, on the core members of this list. These include joy and sadness, hope and fear, joy and pity for another, disappointment and relief, pride and anger, guilt, shame, disgust and many other mental states similar to these (see, e.g., Ortony et al., 1988). Because most of these mental states are subjectively experienced as either pleasant (e.g., joy, pride, relief) or unpleasant (e.g., sadness, fear, disappointment), having a definite hedonic tone has often – from Külpe (1893) to Ortony (2022) – been regarded as the decisive, or at least a central, criterion for being an emotion. The presence of a hedonic tone also justifies subsuming sensory pleasures and displeasures (the pleasant and unpleasant feelings evoked by colors, sounds, tastes, smells etc.) under the category of emotions, despite the fact that they differ from prototypical emotions in other respects (in particular, they have a less complex cognitive basis; see Ortony et al., 1988; Reisenzein, 2009). However, although having a definite hedonic tone may be sufficient for a mental state to qualify as an emotion, it is not universally regarded as necessary: Some theorists also regard certain mental states as emotions, or as emotion components, that do not appear to meet the hedonic criterion. Examples are surprise (e.g., Ekman, 1992; Reisenzein et al., 2019) as well as feelings of arousal (calm vs. aroused), which several emotion theorists regard as a second basic feeling component of emotions (e.g., Wundt, 1896; Russell, 2003).

Disagreement about the classification of a mental state as an emotion is, however, not a hindrance to measuring its intensity, as long as it has an intensity at all. Nor is a worked-out scientific theory of an emotion needed to measure its intensity, at least for the self-report based measurement methods that are the focus of this article (Section 2.2): As long as the emotion whose intensity one wants to measure is known to common-sense psychology (Heider, 1958), it can be targeted in the measurement process by specifying it to competent language users with an appropriate, generally understood emotion term (e.g., “pleasure,” “disgust,” “fear,” “relief”).

### 2.2 Measuring emotion intensity by self-report: direct versus indirect scaling methods

In this article, we focus on psychometric scaling methods for the measurement of emotion intensity. These methods are ultimately based on introspection, and usually take the form of highly structured self-reports. To justify our focus on these methods, it would be sufficient to point out that they are the most frequently used methods for measuring the quality and intensity of emotions. But there are also important theoretical reasons for focusing on introspection-based methods of emotion measurement.

First, there is currently no objective indicator of emotions, whether physiological or behavioral, that can distinguish as finely between the different qualities and intensities of emotions as introspection-based self-reports can (see, e.g., Mauss and Robinson, 2009; Reisenzein et al., 2014). Second, and more fundamentally, introspective self-reports of emotion can claim epistemic priority over other emotion measurements. Even if one assumes that emotional states comprise more than just emotional experience, or that emotions can sometimes be unconscious (e.g., Plutchik, 1989), it is difficult to deny that the primary criterion for the presence, quality, and intensity of an emotion in a target person is the person’s experience, to which the experiencer, and only the experiencer, has direct access. Indeed, it can be argued that the epistemic priority relation between introspective self-reports and other measures of emotions is inextricable: The science of emotion must accord epistemic priority to self-reports of emotional experience to maintain contact with the common-sense understanding of emotional states and their ascription (e.g., Heider, 1958; Laucken, 1974).

Although these arguments, particularly the second one, often evoke the dissent of emotion researchers when presented openly, they appear to be widely accepted implicitly. This is evidenced by the fact that self-report based measurements are typically used as the “gold standard” for validating behavioral and physiological measures of emotion (e.g., Reisenzein et al., 2014), and for selecting or constructing stimuli to induce emotions in laboratory studies (see also Kron, 2019).

The traditional aim of psychometric scaling methods has been to assess presumed mental quantities, such as sensations or emotional feelings, on a metric (interval or higher) scale level. In this article, we are only concerned with such attempts at metric measurement, i.e., measurement in the classical sense (Michell, 1999). The scaling methods that have been proposed for this purpose are often divided into “direct” and “indirect” methods (e.g., Engen, 1971; Sixtl, 1982; Marks and Gescheider, 2002). This distinction will also be used here because of its fundamental importance. The most important difference between direct and indirect scaling methods is how much of the process of constructing a metric scale is trusted to the subject.

#### 2.2.1 Direct scaling methods for measuring emotion intensity

Direct scaling methods, when proposed for metric measurement, are based on the assumption that humans are in principle able to provide metric measurements of the intensity of their sensations and feelings, which can then be more or less directly used in subsequent data analyses. Direct scaling methods fall into two main classes, corresponding to the two main metric scale levels, interval and ratio, that their proponents believe can be attained with them (e.g., Engen, 1971).

The “intended interval-scale” methods, sometimes called *partition methods* (following Stevens, 1975), assume that people are able to partition the latent intensity continuum into a set of equal-sized intervals (e.g., Engen, 1971; Marks and Gescheider, 2002). The most prominent partition method is the category rating scale (Guilford, 1954). As mentioned, the quality-intensity emotion rating scale, or at least certain versions of this scale (Guilford, 1954; Anderson, 1981), are examples of this direct scaling method.

Despite their ubiquity and easy of use, rating scales in general have been extensively criticized (e.g., Baumgartner and Steenkamp, 2001; Marks and Gescheider, 2002; Yannakakis and Martínez, 2015; Uher, 2018, 2023), and these criticisms are also relevant for emotion rating scales (see also Lim, 2011). Probably the most important actual or potential problems of rating scales for the measurement of emotion intensity are (1) their limited resolution (see, e.g., Böckenholt, 2004); (2) their comparatively large contamination with random error, given typical and realistically possible conditions of use (see Section 3.4); (3) their nonmetric scale level, and, partly responsible for it (4) their susceptibility to stimulus and instructional context effects, as well as to diverse response biases (e.g., Poulton, 1989; Baumgartner and Steenkamp, 2001), such as the tendency to avoid the extremes of the scale (Stevens and Galanter, 1957). It should be noted, however, that there are ways to reduce context effects and response biases (Anderson, 1982) and that the influence of some commonly claimed rating response styles, such as acquiescence (Baumgartner and Steenkamp, 2001), on emotion intensity ratings appears to be minimal in typical assessment contexts (see Schimmack et al., 2002).

The “intended ratio-scale” class of direct scaling methods comprises various forms of *magnitude scaling*, which gained prominence primarily because of S. S. Stevens’ psychophysical research (e.g., Stevens and Galanter, 1957; Stevens, 1975). The most frequently used magnitude scaling method is *magnitude estimation*, where participants are required to judge the ratio of the intensity of a sensation or feeling to an experimenter-supplied or (implicitly) self-chosen comparison standard.

Magnitude scaling methods have become highly popular in the field of sensory measurement (e.g., the measurement of sound intensity or brightness; Marks and Gescheider, 2002), not last because they were advertised as superior to category ratings (Stevens and Galanter, 1957; Stevens, 1975). Nonetheless, magnitude scaling methods have only been rarely used for the measurement of emotion intensity (examples are Moskowitz and Sidel, 1971; Sullivan, 1971; Teghtsoonian and Frost, 1982; Galanter, 1990; see also Cardello and Jaeger, 2010; Lim, 2011). The main reason for this neglect may have been practical: As Cardello and Jaeger (2010) and Lim (2011) point out for the field of sensory science, some participants have difficulties learning magnitude estimation procedures, and the resulting data are more cumbersome to process than ratings. In addition, the claimed advantages of magnitude scaling over category ratings—that magnitude scaling is immune to stimulus and instructional context effects, and yields a ratio scale (Stevens, 1975)—have turned out to be highly questionable (see Anderson, 1981; Birnbaum, 1982; Ellermeier and Faulhammer, 2000; Masin, 2022).

#### 2.2.2 Indirect scaling methods for measuring emotion intensity

The criticisms of the direct scaling methods are good reasons to consider *indirect scaling methods* as alternative methods for measuring emotion intensity. Two common arguments for indirect scaling methods are that they are less susceptible to response biases (see, e.g., Brown and Maydeu-Olivares, 2018) and that they yield more precise measurements than direct scalings. Historically, however, the central motivation for developing indirect scaling methods was the belief that direct scaling methods cannot provide metric measurements, whereas indirect scaling methods can.

The most conservative indirect scaling position is that people’s introspective abilities are limited to judging the intensities of the sensations or feelings evoked by different stimuli on an ordinal scale, i.e., as greater, equal or less (e.g., Fechner, 1860, 1871; Thurstone, 1927). A more optimistic view, apparently first articulated by Plateau (1872), is that people can additionally order *differences* between feeling intensities. We come back to this assumption in Section 3.5. The important point at present is that, in both cases, the introspecting subject is assumed to be only able to operate on the ordinal level of measurement: to rank-order intensities, or to (also) rank-order intensity differences. It is the researcher, who—on the assumption that the ordinal judgments are based on a latent quantitative variable—attempts to infer the exact levels of this variable from the ordinal judgments. This is achieved by using a scaling model (e.g., Thurstone, 1927; Boschman, 2001; Marley and Louviere, 2005). Interpreted from the realist view of mental measurement (see Sections 3.6 and 5.2), a scaling model is a theory about how (by which cognitive processes) the person’s overt judgments are constructed on the basis of—in the case of conscious mental states—her introspective observation of the latent quantity. The process of estimating scale values attempts to invert the hypothesized judgment process, i.e., to estimate the values of the latent variable from the ordinal data plus the scaling theory’s assumptions about the judgment process. An example of an indirect scaling model, the ODS model, is described in Section 3.6.

As said, psychometric emotion measurement is today dominated by a direct scaling method, the quality-intensity emotion rating scale. But this was not always so. To the contrary, at the beginnings of psychology as an academic discipline in the 19th century, indirect methods of measuring sensations and feelings predominated. The reason was that most psychologists of this period, despite regarding introspection as psychology’s main method, did not believe that the intensity of sensations and feelings can be *directly* scaled. The first application of an indirect scaling method to emotional experience was made by Fechner (1871), who proposed an early version of best-worst scaling (see Cardello and Jaeger, 2010) to measure the aesthetic pleasantness of geometric figures. Somewhat later, Cohn (1894) used the paired comparison method (see Thurstone, 1927) to measure the pleasantness of colors, and Titchener (1902) extended the method to the measurement of the basic feelings postulated in Wundt’s (1896) tri-dimensional theory of emotions. These early applications of indirect scaling methods to emotions were not based on an explicit scaling model; instead, scale values were estimated using intuitively plausible, simple calculations, such as counting how often each stimulus is judged as more pleasant than others. It was left to Thurstone (1927) to supply one of these methods, the paired comparison procedure, with an explicit statistical judgment (scaling) model that promised to yield metric measurements, provided that its assumptions are met. Thurstone’s (1927) publication led to a rapid increase in the use of the paired comparison method for measuring attitudes, values and hedonic feelings (Cardello and Jaeger, 2010).

Although direct scaling methods for measuring emotional feelings, in the form of the category rating scale, also have a long history (Major, 1895), they gained popularity only in the 1940ies and 1950ies (Lorr, 1989; Cardello and Jaeger, 2010). They were first utilized more widely in the field of sensory hedonics, where the so-called *9-point hedonic scale*, a bipolar labeled category scale ranging from “dislike extremely” to “like extremely” (Peryam and Pilgrim, 1957) became dominant (Cardello and Jaeger, 2010). The main reason for its rise in popularity was practical: For measuring people’s hedonic reactions to foods, beverages etc., the paired comparison method was experienced as too cumbersome or even inapplicable (Cardello and Jaeger, 2010).

It was only in the mid-1950ies to early 1960ies that researchers became interested in the assessment of specific emotions and moods (e.g., Nowlis and Nowlis, 1956; see Lorr, 1989). When they did, they turned to the quality-intensity rating scale almost by default. The main reason was again most likely practical: Rating scales are well-suited for the quick and comprehensive assessment of a person’s momentary emotions or moods, which was then a major research interest (Nowlis and Nowlis, 1956). Still, it is worth noting that indirect scaling methods were not even considered anymore when emotion researchers began to measure specific emotions. From the beginning, nearly all attempts to measure specific emotions have used direct scaling methods—essentially some version of the ubiquitous quality-intensity rating scale.

#### 2.2.3 More recent developments

Over the past two decades, the firm grip of the classical rating scale on emotion measurement has begun to loosen a little, due to the emergence of several new or improved direct and indirect scaling methods. Perhaps the most noteworthy development in the direct scaling camp is a new type of labeled intensity rating scale, where the placement of the intensity labels is determined empirically through magnitude estimation. These scales are known as *labeled affective magnitude scales* (e.g., Schutz and Cardello, 2001; Lishner et al., 2008; for reviews, see Cardello and Jaeger, 2010; Lim, 2011; Schifferstein, 2012; Ares and Vidal, 2020). Although it seems that these scales have so far only been used to measure the intensity of pleasure and displeasure, they could easily be adapted to assess specific emotions.

In the indirect scaling camp, too, new methods have been proposed to measure emotion intensity. Particularly noteworthy is *Best-Worst Scaling*, a modern probabilistic version of the scaling procedure proposed by Fechner (1871) (Finn and Louviere, 1992; for more recent accounts, see Marley and Louviere, 2005; Jaeger et al., 2008; Louviere et al., 2015). This scaling method has become increasingly popular during the past years for measuring preferences and attitudes in several disciplines (see Schuster et al., 2024) and has also been utilized to measure emotions. So far, the focus of Best-Worst scaling in this area has been the measurement of sensory pleasure and displeasure (e.g., Jaeger et al., 2008; Jaeger and Cardello, 2009; Mielby et al., 2012; see also Cardello and Jaeger, 2010), but it has also been used to measure the intensity of fear (Farkas et al., 2021) and to scale the intensity of positive and negative emotions expressed in text (Mohammad and Bravo-Marquez, 2017). We believe that the probabilistic difference scaling methods advocated in this article (see Section 3) represent an even more effective indirect scaling alternative for measuring the intensity of emotions.

## 3 Difference scaling methods

### 3.1 Difference data

Difference scaling methods are indirect, unidimensional scaling methods based on difference data. Difference data (in our case, judgments) come in two main kinds: direct difference comparisons or *quadruple judgments* (QCs), and *graded paired comparisons* (GPCs). Both judgment tasks are special forms of the paired comparison method. In the QC task—the classical difference judgment task—the participants are in each trial presented with two pairs of stimuli (*a*, *b*) and (*c*, *d*) and indicate which pair differs more on the judgment dimension. For example (Junge and Reisenzein, 2015, Study 1), participants are shown two pairs of disgusting pictures side by side on the screen, and are asked to indicate in which pair the stimuli differ more in the intensity of evoked disgust.

In contrast, in the GPC task, two stimuli *a* and *b* are compared, as in the classical paired comparison task (e.g., Cohn, 1894; Thurstone, 1927). However, different from classical paired comparisons, the participants indicate not only which stimulus has the larger value on the judgment dimension, but also how much greater the difference is. Importantly, nonmetric scaling methods for GPCs assume that these judgments have only an ordinal scale level. So understood, the GPC task can be seen as a combination of the classical paired comparison task with an ordinal rating of differences. To illustrate, in another part of their Study 1, Junge and Reisenzein (2015) presented participants with the disgusting pictures in pairs and asked them to indicate which picture was more disgusting, as well as how much more disgusting it was, on a response scale with six ordered categories ranging from “just barely noticeably more” to “extremely more.”

### 3.2 Scaling models for difference data

For both QCs and GPCs, a number of scaling methods are available. Here, we only consider nonmetric methods. In the first empirical studies using difference scaling, unidimensional versions of nonmetric multidimensional scaling were used (see, e.g., Schneider, 1982 for QCs and Orth, 1982, for GPCs). A disadvantage of these methods is, however, that they are not based on a statistical model (for additional discussion, see Haghiri et al., 2020). This drawback has been rectified in more recent, probabilistic scaling models whose main varieties are *Maximum Likelihood Difference Scaling* (MLDS) for QCs (Maloney and Yang, 2003; Knoblauch and Maloney, 2008), and *Ordinal Difference Scaling* (ODS) for GPCs (Agresti, 1992; Boschman, 2001; see also Tutz, 1986). These two scaling methods are actually closely related in terms of their basic assumptions (see Junge and Reisenzein, 2015). Furthermore, because both methods were developed for the scaling of ordinal difference data, both can claim to be founded on an axiomatic measurement theory developed for such data, the difference measurement model (Krantz et al., 1971). This means that ODS and MLDS not only allow to estimate precise scale values and to determine the overall fit of the model to the data, but also to construct a statistical test of the crucial axioms of difference structures that need to be fulfilled to obtain a metric scale (see Section 5).

### 3.3 Advantages of ODS over MDS

Although both MLDS and ODS are suitable for the measurement of emotion intensity (Junge and Reisenzein, 2015), in our studies we focused on ODS of GPCs, because this method has several advantages over MLDS, particularly for emotion measurement (Junge and Reisenzein, 2015). Most importantly, ODS is more economical than MLDS, because it needs much fewer input data (for details, see Junge and Reisenzein, 2015; and Schneider, 1982). This is a direct consequence of the fact that the input data of ODS (i.e., GPCs) require comparing pairs of stimuli, whereas those of MLDS require comparing pairs of pairs. The savings in the number of paired comparisons enabled by GPCs are substantial and increase with the number of stimuli (see Junge and Reisenzein, 2015). Additionally, because the GPC task requires processing only two stimuli rather than four in each trial, as the QC task does, it is arguably less cognitively taxing for the participants (Junge and Reisenzein, 2015). Finally, MLDS in contrast to ODS requires that the rank-order of the stimulus intensities is known, which in the case of affective stimuli usually means that this rank order has to be separately estimated for each participant prior to the QC task.

Importantly, the economical advantage of ODS does not come at the expense of lower-quality scalings: Junge and Reisenzein (2015) found that ODS scalings of GPCs were at least as reliable, and correlated at least as highly with direct ratings of emotion intensity, as MLDS scalings of QCs of the same stimuli. Hence, ODS can be regarded as an economical alternative to MLDS for the difference scaling of emotion intensity.

### 3.4 Differences to classical Thurstonian scaling

Although ODS and MLDS stand in the tradition of Thurstonian scaling models (Thurstone, 1927; Böckenholt, 2006), they differ in a crucial respect from other models of this class, including best-worst scaling (Marley and Louviere, 2005): They use not only information about the ordering of stimulus intensities, but also about the ordering of intensity differences. This additional information leads to several advantages of difference scaling methods (see Knoblauch and Maloney, 2008, for the case of MLDS; and also Anderson, 1981) that we here illustrate by comparing them to Thurstone’s (1927) classical paired comparison model. First, in contrast to the Thurstonian model, difference scaling models allow to test measurement axioms required for a metric representation (Krantz et al., 1971; see Section 5). Second, they allow to scale stimuli with clear suprathreshold intensity differences, i.e., stimuli that are perfectly discriminable, whereas the Thurstonian model can only estimate distances between stimulus pairs that are close enough to be not consistently distinguishable. Third, the difference scaling models allow to scale the data of individual participants, because a single judgment of the stimulus pairs or quadruples is sufficient to obtain reliable scale estimates. In contrast, Thurstonian scaling of individual data is unfeasible for many kinds of stimuli, because it requires numerous repetitions of the paired comparisons to obtain reliable estimates of the confusion probabilities (Anderson, 1981). Fourth, in the Thurstone model, the obtained scale depends crucially on the assumed error distribution, whereas MLDS has been found to be robust to variations of the error distribution (Maloney and Yang, 2003), and we have found the same for ODS in additional analyses of our data. Finally, whereas the interpretation of intervals on the MLDS and ODS scales as intensity differences is transparent, an analogous interpretation of the intervals on the confusion-based Thurstone scale requires additional assumptions (Knoblauch and Maloney, 2008).

### 3.5 Are people able to order intensity differences?

The information that difference scaling methods attempt to elicit from participants was first described by Plateau (1872) in a seminal paper on the measurement of sensations. In this article, Plateau (1872) conjectured:

“When we experience, either simultaneously or successively, two physical sensations of the same sort, but of different intensities, we can easily judge which of the two is the stronger and, we can, moreover, decide whether the difference between them is great or small. But there, it seems, the comparison must end…we appear to be incapable of estimating the numerical ratio between the two intensities of two sensations in this way” [Plateau, 1872, translation by Laming and Laming (1996); p.136]

Note that the GPC task nearly precisely matches Plateau’s (1872) description of what humans are, in his view, able to provide: Information about the ordering of the intensity of the compared sensations or feelings, and information about the ordering of their intensity differences (“barely different,” “moderately different,” “very different” etc.). Note also that Plateau’s (1872) views on people’s judgment abilities provide a precise explanation of the intuition behind the commonly made claim that rating scales are somewhere between the ordinal and metric scale levels, i.e., that they contain more than ordinal information, even though not metric information: People are also able to order the intensity differences between different sensations or feelings.

Are Plateau’s assumptions plausible, and hence, can difference scaling work in principle? His first assumption, that people can reliably rank-order the intensity of the sensations or feelings evoked by different stimuli, is largely uncontroversial, provided that the intensity differences are not too small. However, for GPCs, this assumption can also be checked by testing the transitivity of the dichotomized GPC judgments. For the GPCs of emotion intensity collected in our studies, this analysis (conducted for the present article) revealed that the judgments were nearly perfectly transitive for practically all participants.

Thus, the validity of the GPC (and, analogously, the QC) method depends on Plateau’s (1872) second assumption, that people are also able to consistently order intensity differences. As discussed in Section 5, this is still not enough; the ordering of intensity differences must also fulfill an additivity condition. However, already the more basic ability to order intensity differences has been questioned by some authors. Specifically, in the field of preference measurement, where axiomatic difference measurement has been a major research topic (for reviews, see, e.g., Krantz et al., 1971; Farquhar and Keller, 1989; Köbberling, 2006; Moscati, 2019), some researchers have doubted that people are able to compare and order preference differences (e.g., Machina, 1981). However, other researchers in this field have argued that this doubt is unfounded, that people are well able to order preference differences, and that the obtained data make sense (von Winterfeldt and Edwards, 1986). In any case, there is empirical evidence that people are able to provide reliable judgments of intensity differences of *sensations* and *emotional feelings* (e.g., Schneider, 1982; Knoblauch and Maloney, 2008; Junge and Reisenzein, 2015).

While these data are ultimately decisive, to convince oneself that people are indeed able to order the size of emotion intensity differences, it is best to consider an example (see also Krantz et al., 1971, p. 140–141, and von Winterfeldt and Edwards, 1986, pp. 209–210, who discuss similar examples). Imagine you are shown three affective pictures *a*, *b*, *c*, and find that they evoke, in order, just noticeable pleasure (say 1 on a 0–10 rating scale), mild pleasure (3), and very strong pleasure (9). As mentioned in the introduction, such intensity judgments of emotion are commonly made in everyday life, although not usually on a rating scale. Then ask yourself whether you would be willing to say that the difference between *b* and *c* (between mild and very strong pleasure) is greater than that between *a* and *b* (just noticeable and mild). If you answer yes (as we do), you agree that intensity differences of pleasure can be rank-ordered.

### 3.6 ODS as a psychological measurement theory

#### 3.6.1 The ODS model

On a realist interpretation of measurement (see Section 5), the statistical model underlying ODS is a small psychological theory of the mental processes that underlie responses in the GPC task. (The same is true for the MLDS model of the QC task; see Junge and Reisenzein, 2015). The ODS model can be summarized in two equations:


(1) $$\Delta_{a,b}=\Psi_{b}-\Psi_{a}+\varepsilon ,\mathrm{with}\;\varepsilon ∼Ν\left(0,\;\;\sigma^{2}\right)$$

(2) $$\begin{array}{l} R_{a,b}=j\;\;\mathrm{if}\;\theta_{j-1}<\Delta_{a,b}\leq \theta_{j},\mathrm{with}\;j=1,\ldots ,J\;\mathrm{and}\\ -\infty =\theta_{0}<\theta_{1}<\ldots <\theta_{J-1}<\theta_{J}=+\infty \end{array}$$

Ψ_a_ and Ψ_b_ are the scales values of the two stimuli *a* and *b* compared in a trial of the GPC task, and Δ_a,b_ is an internal decision variable on which the overt response R_a,b_ is based. In addition, the ODS model contains θ_1_, …, θ_J-1_ unknown thresholds separating the response categories, which, like the scale values, must be estimated.

Interpreted in terms of mental processes, and illustrated for emotion intensities, the ODS model can be described as follows. Equation 1 describes the initial stimulus representation and comparison process. It assumes: (1) the emotion intensities evoked by the two stimuli *a* and *b* presented to the participant in a trial of the GPC task give rise to two emotion intensities whose values are on average Ψ_b_ and Ψ_a_. (2) The emotion intensities are compared, either simultaneously or successively, by a process that (implicitly) computes the difference between them (see 3.6.2 for an explication of this process). (3) Both processes (the elicitation of the feelings and their comparison) are biased by independent random noise stemming from a normal distribution with constant variance σ^2^. Note, however, that the distributional assumption can be changed, and the constant variance assumption can in principle be relaxed.

Equation 2 describes the response process. It assumes: (4) The decision variable Δ_a,b_, which represents the computed difference between the intensities of the emotions elicited by stimuli *a* and *b* in a given trial, is mapped into category j of the response scale consisting of J ordered categories, whenever Δ_a,b_ lies between the thresholds θ_j-1_ and θ_j_ that mark the boundaries of j on the latent continuum. If the judgment noise were zero, the difference between the two intensities would be exactly mapped into the correct response category; however, because of the presence of random noise, another response category will occasionally be chosen, and this will happen more frequently, the closer the intensities evoked by the two stimuli are on the judgment dimension.

The aim of ODS scaling is to estimate, from the observable responses R_ab_ (the ordinal graded comparisons of stimuli *a* and *b*), the latent scale values of the stimuli assumed to underlie these responses.

As just described, the ODS model is a special case of the ordered (or cumulative) probit model (McKelvey and Zavoina, 1975; Greene and Hensher, 2010), which can be obtained in a straightforward manner by applying the ordered probit model to GPCs (Agresti, 1992; Boschman, 2001; as pointed out by Agresti (2010), the proportional odds assumption characteristic for cumulative link models is implied by a simple latent variable model). The scale values and thresholds can be estimated using maximum likelihood methods with widely available software. For example, in R (R Core Team, 2023), one can estimate the ODS model parameters with the functions *polr* in library *MASS* and *clm* in library *ordinal* (Christensen, 2018). Functions for the Bayesian estimation of the ordered probit model are also available (e.g., Gelman and Hill, 2006; Bürkner, 2017). In our research with ODS, we estimated the ordered probit model using a bias-reducing version of maximum likelihood estimation, *bpolr* (Kosmidis, 2014). This was done to avoid issues of separation, an estimation problem that can occur particularly with sparse data, e.g., when estimating the model for individual subjects (for more information, see Junge and Reisenzein, 2015).

#### 3.6.2 Possible elaborations of the ODS model

As it stands, the ODS model is a relatively coarse and abstract theory of the mental processes that take place in the GPC task. Elaborations of the model are possible, however, two of which we sketch here.

First, one could refine the ODS model by distinguishing between assumed subprocesses. In particular, one could introduce a threshold for noticing intensity differences, and one could try to tease apart the different sources of random noise that contribute to the error term and model them by separate parameters. These noise sources are in particular (a) trial-by-trial fluctuations of the emotion intensities evoked by a stimulus (e.g., because of different degrees of attention devoted to the stimulus in different trials); (b) fluctuations due to the limited precision of the difference comparison mechanism; (c) fluctuations in the mapping of the decision variable to the response categories; and (d) response errors due to lapses of attention or wrong key presses. This general path to model elaboration has been taken in other areas of psychometric modeling, for example in models for temporal order and simultaneity judgments (e.g., García-Pérez and Alcalá-Quintana, 2012; see also Reisenzein and Franikowski, 2022). Its practical advantage for measurement is that, by isolating the different component processes and estimating them separately, purer estimates of the latent emotion intensities can be obtained.

Second, one could elaborate the ODS model into a full-fledged cognitive process model, that is, a representational-computational model of the judgment process. This requires specifying the underlying representation medium or media and the basic operations performed with these representations during the judgment process. A computational model does not at present exist for GPC (nor QC) judgments. However, Petrov and Anderson (2005) have proposed a computational model for category ratings in the well-researched ACT-R cognitive architecture (e.g., Anderson, 1983; Anderson and Lebiere, 1998). This computational model, which combines the Thurstonian theory of category ratings (Torgerson, 1958) with the theory of memory incorporated in the ACT–R architecture, could serve as the template for an analogous computational model of the GPC task. We briefly sketch here how this model might look like, because doing so adds substance and plausibility to our realistic interpretation of ODS as a psychological judgment theory.

Following analogous assumptions by Petrov and Anderson (2005) for the category rating task, we begin by assuming that the first step of the GPC task is the creation of emotion intensities for the two stimuli *a* and *b* compared in a trial. The details of this process need not be specified for measurement purposes, with one exception: We assume that these intensities are a form of analog representation of magnitudes (see Beck, 2015, for more on this concept). The two intensity representations are then processed within the central subsystem of ACT-R. The first central processing step is the computation of the intensity differences. We propose that this is achieved by a subpersonal similarity matching process, as implemented in the ACT-R architecture; hence it does not require symbolic (propositional) representations. Because the two intensities lie on an unidimensional quality continuum, the similarity comparison process amounts to a comparison of the intensities of the emotions (see already Thurstone, 1927). Furthermore, we submit that the resulting difference representation is again nonpropositional: It is an analogical representation of perceptual closeness or distance subjectively experienced as a feeling of smaller or greater difference.

This difference representation is next compared by the partial matching mechanism to a set of memory anchors that encode prototypical degrees of intensity differences more or less specific to the emotion in question (see Petrov and Anderson, 2005). More precisely, the difference representation activates an anchor whose magnitude is similar to the computed intensity difference. Anchor selection is stochastic and also depends on other factors besides similarity, such as recency and base-level strength. Furthermore, following once more Petrov and Anderson (2005), we may assume that, if there is a large discrepancy between the difference representation and the magnitude of the anchor retrieved from memory, an explicit correction mechanism may increment or decrement the response suggested by the anchor. Finally, one could include a learning mechanism that causes slight changes of the magnitude of the anchor that corresponds to the response in this trial (Petrov and Anderson, 2005).

### 3.7 Estimating the zero point

Unless special measures are taken, ODS—like all comparative judgment methods (Guilford, 1954; Böckenholt, 2004)—does not estimate the zero point of the scale. However, for many research questions of emotion psychology, it is at least advantageous, if not necessary, to also know the natural zero point (the absence of emotion), and thus to have available not just an interval scale (see Section 5) but a ratio scale. For example, a ratio scale of emotion intensity is needed for stringent tests of quantitative emotion models (e.g., Junge and Reisenzein, 2013, Study 1).

In our studies, we estimated the zero point using simultaneously collected direct ratings of emotion intensity. These ratings were made on numerical scales anchored at the lower end by the natural zero point of emotion intensity (e.g., “the picture evokes no pleasure”) and at the upper end by “extremely intense.” To locate the zero point on the ODS scale, we then transformed the ODS scale values into the range of each participant’s ratings. Note that this method of estimating the zero point only relies on the ratings for estimating the distance from zero of the lowest-intensity stimulus. The error of this estimate will be minimal if that stimulus is indeed close to zero (i.e., if a low-intensity stimulus is in the set), which was almost always the case in our studies. However, it is also possible to estimate the zero point as part of the difference scaling procedure. The simplest way to achieve this is by including an affectively neutral (at least with respect to the emotion under study) stimulus, such as an affectively neutral picture. Additional methods for estimating the zero point of scales derived from comparative judgments are discussed by Guilford (1954) and Böckenholt (2004).

While the natural zero point of emotion intensity is the same for different people, to optimize the interpersonal comparability of emotion intensity scales, it would be ideal to also have an interpersonally comparable scale unit. For some research questions, this is even necessary (see, e.g., Bartoshuk et al., 2005; Luce, 2010; Schifferstein, 2012). A fully satisfactory solution to this problem does not exist. However, a pragmatic solution is to fix the scale unit by using an approximately consensual end-point anchor label, such as “maximal” or “extremely,” on a parallel rating scale (see Borg, 1962; Marks et al., 1983). This approach is, in fact, common practice for labeling emotion rating scales. Sometimes, in particular when using imagined emotion-evoking scenarios, it is also possible to include a stimulus into the difference scaling procedure that can be assumed to evoke near-maximum emotion intensity in most people (Reisenzein and Junge, 2024). Another possibility may be to fuse difference scalings with data from cross-modality matching (Bartoshuk, 2014).

## 4 Measuring emotion intensity with difference scaling methods

In our studies, participants made GPC judgments of the intensities of a broad range of emotions: pleasure and disgust evoked by affective pictures, amusement and surprise induced by quiz items, relief and disappointment about lottery outcomes, hope and fear, disappointment and relief experienced in diverse imagined scenarios, and anger and pity in hypothetical helping situations (Junge and Reisenzein, 2013, 2015, 2016; Reisenzein and Franikowski, 2019; Reisenzein and Junge, 2024). In all studies, the participants also made direct scalings of emotion intensity on 0–10 or 0–100 numerical rating scales ranging from “not at all” to “extremely”; in one case, a combination of rating and ranking (Kim and O’Mahony, 1998) was used. In the studies reported in Junge and Reisenzein (2016), we additionally collected QC judgments, i.e., direct comparisons of intensity differences.

The GPC judgments were scaled with ODS and/or, in some cases, with MLDS, taking advantage of the fact that GPCs can be expanded to QCs, the data needed for MLDS (Junge and Reisenzein, 2013, 2015; see Section 5). The difference scaling models were fitted to the data of the individual participants and the estimated scale values were linearly transformed into the range of the rating scale to estimate the zero and an extreme point, and thus, improve the interpersonal comparability of the measurements.

### 4.1 Reliabilities and discrimination capacity

Across the studies conduced by Junge and Reisenzein (2013, 2015, 2016), the difference scalings of the individual participants had an average reliability (estimated either by repeated measurements, or a bootstrap procedure) of *r* = 0.95. In contrast, the average reliability of the ratings (estimated as the re-test correlation between ratings made before and after the GPCs, or in two different sessions) was *r* = 0.79. Furthermore, whereas the 0–10 category rating scale used in most of our studies allowed the participants to distinguish, at best, between one scale point, additional analyses revealed that the difference scale (transformed into the same range) enabled them to reliably distinguish between about 0.5 scale points.

In unpublished research, similar findings were obtained for ODS scalings of hope, disappointment, fear and relief in hypothetical scenarios (Reisenzein and Junge, 2024) and for feelings of pity and anger toward others in helping scenarios (Reisenzein and Franikowski, 2019).

### 4.2 Robustness of GPC scalings to variations of the difference scaling method

Scalings of the GPCs by ODS and by MLDS (after expanding the GPCs to QCs; see Section 5) yielded nearly identical results, with average intra-individual scale intercorrelations of *r* > 0.99 (Junge and Reisenzein, 2015). Additional analyses conducted by us on the data from Junge and Reisenzein (2015) found equally high correlations between the ODS scale values and those estimated by a metric version of difference scaling, additive functional measurement (AFM, Boschman, 2001). This replicates findings by Boschman (2001) obtained for the scaling of sensory attributes. Junge and Reisenzein (2013) obtained slightly lower (average intra-individual *r* = 0.95) correlations between MLDS and AFM scalings. Taken together, these findings support the robustness of the GPC scaling results to variations of the probabilistic difference scaling method.

### 4.3 Testing emotion theories with difference scalings

Junge and Reisenzein (2013) used the MLDS and AFM models as auxiliary measurement theories to test two small psychological emotion theories. The intensities of the emotions were first estimated using difference scaling on the individual level, and these measurements were then used in experimental tests of the emotion theories. This sequential approach (measurement—theory test) corresponds to the classical approach in scaling (see Anderson, 1981) and has been advocated by several authors in the field of structural equation modeling, most recently by Rosseel and Loh (2022), who also discuss its advantages.

In Experiment 1, we tested a quantitative belief-desire model of the intensity of disappointment and relief (Reisenzein, 2009) elicited by unobtained gains and losses in monetary lotteries. Belief and desire strengths were experimentally manipulated by varying, respectively, the objective probability and size of a possible monetary gain or loss (*cf.* Mellers et al., 1997). Nonlinear regression was used to fit the quantitative emotion models to the data of the individual participants, and the squared correlation between predicted and measured emotion intensity was used as the index of global model fit. For details, readers are referred to the original article (Junge and Reisenzein, 2013).

High fits of the emotion models were obtained for the indirect scales of most participants: *R*^2^ was >0.90 for 68% of the participants if the MLDS scale values were used as the dependent variable, and for 90% if the AFM scale values were used. The explained variance in emotion intensity is so high that one may conclude that beliefs plus desires are sufficient causes of the intensity of relief and disappointment, as the tested emotion models assume. Furthermore, the pattern of scale values corresponded to the predicted pattern of a (nonlinear) fan for nearly all participants. In contrast, if emotion intensity ratings (the mean of two repeat measurements) were used as the dependent variable, only 13% of the participants attained an *R*^2^ > 0.90 for relief and only 38% for disappointment. In addition, a separate test of the predicted linear interaction effect of the experimental manipulations on emotion intensity, reliably detected this interaction for the difference scales, but missed it for disappointment if the direct ratings were used. Incidentally, the better performance of the AFM scalings in this as well as the second study by Junge and Reisenzein (2013) might mean that GPCs contain more than just ordinal information about intensity differences.

In the second study, Junge and Reisenzein (2013, Experiment 2) tested a theory of (some) determinants of the intensity of disgust. Disgusting pictures were experimentally varied in size (big or small) and coloration (normal colored or false colored). Based on evolutionary considerations, it was predicted that the two manipulations would have an additive or superadditive effect on emotion intensity. Again, the difference scalings revealed the predicted pattern for the majority of the participants. For example, pooled across four experimentally manipulated disgust pictures, 51% of the participants conformed to the disgust model for the MLDS scalings and 85% for the AFM scalings, but only 30% did so for the ratings (made only once in this study, but after the GPC task).

These findings are important because they demonstrate the scientific utility of the indirect scaling methods. Experiment 1 showed that difference scalings of emotion intensity, but not direct intensity ratings, allowed to obtain support for quantitative emotion theories on the level of the individual subjects (Junge & Reisenzein, Study 1). Because most theories in psychology are formulated on the level of the individual, this is the level on which they should be preferably tested—a methodological recommendation repeatedly given (see, e.g., Estes, 1956; Woike et al., 2023) but still too rarely followed, particularly in emotion research. Experiment 2 demonstrated the same point for tests of ordinal causal hypotheses (Junge and Reisenzein, 2013, Exp. 2). Furthermore, the experiments demonstrated that difference scalings increase the power of statistical tests on both the individual and group levels. For example, they allowed to reliably detect predicted interaction effects, which are often missed with direct ratings (e.g., Nagengast et al., 2011).

### 4.4 Two reasons for the superior performance of difference scalings

One reason for the superior performance of the indirect scales compared to direct ratings in the reported tests of emotion models is their greater precision. This is in part simply a consequence of the fact that the indirect scales were based on a much larger set of judgments (although it should be noted that each GPC judgment provides only information about the difference between two emotion intensities). It could therefore be argued that, instead of using GPCs, one could simply replicate stimulus ratings more often and average them. This is standard practice in direct scalings of sensations of individual subjects, where the stimuli are presented numerous times (e.g., 50 times in Montgomery, 1982). However, apart from the fact that this does not address the limited resolution of ratings nor improve their scale level, numerous repeated ratings are usually not possible for affective stimuli (see also, Anderson, 1981). The main reason is that most emotional stimuli (e.g., affective pictures) are easy to memorize and participants could therefore simply reproduce their previous ratings. Aggregating ratings across participants to increase reliability is also of limited usefulness, because there are often large interindividual differences in emotional reactions to the same stimuli. Finally, the use of multiple indicators to increase the reliability of emotion ratings (e.g., Kline, 2016) is restricted, among other factors, by the fact that for many emotions, it is difficult to find more than a few emotion terms that have sufficient semantic similarity (e.g., what would be good multiple indicators for relief or disappointment?).

A second reason for the superior performance of the difference scales in our tests of emotion theories (Junge and Reisenzein, 2013) could have been that they approximated the metric scale level better than the ratings. This issue is addressed next.

## 5 Testing measurement axioms

As mentioned in the introduction, our approach to measurement combines the modern psychometric (i.e., latent-variable) approach to measurement, in our case represented by probabilistic difference scaling models, with the representational theory of measurement (RTM; e.g., Suppes and Zinnes, 1963; Krantz et al., 1971). This combination is facilitated by the fact that an axiomatic measurement theory for difference data—the data that constitute the input to the difference scaling models—exists (Krantz et al., 1971, Ch. 6). However, in our view, the integration of the latent-variable and RTM approaches to measurement requires a non-standard interpretation of RTM. To make clear where we differ from the standard interpretation of RTM, we briefly summarize it first.

### 5.1 The standard representation of RTM, illustrated for difference structures

The main goal of RTM is to specify the conditions, formulated as axioms, that the qualitative (typically, ordinal) relations among the levels of a variable must fulfill to allow a homomorphic (structure-preserving) mapping into a subset of the numbers, usually the reals. In the case of difference measurement, the qualitative (ordinal) structure is <*A* × *A*, ≿ > and the numerical structure is <ℝ, ≥ >. For example, in difference measurement of emotion intensity, *A* is a set of affective stimuli, *A* × *A* is the set of stimulus pairs (*a*, *b*) from *A*, and ≿ is the ordering of perceived differences in intensities of the feeling evoked by pairs of stimuli (*a*, *b*) in a difference judgment task. The most direct way of obtaining these difference comparisons is the QC task (Section 3.1); however, they can also be retrieved from GPCs, as follows (Roberts, 1979; Orth, 1982): For all pairs of stimulus pairs (*a*, *b*; *c*, *d*), *ab* ≻ *cd* (the intensity difference between the feelings elicited by *a* and *b* is greater than that between the feelings elicited *c* and *d*) if GPC(*a*, *b*) ≻ GPC(*c*, *d*) (example: *a* is judged as eliciting *much more* pleasure than *b*, while *c* is judged as eliciting *somewhat* more pleasure than *d*). If the two GPC judgments are equal, one is randomly chosen to be greater.

The axioms of difference structures impose constraints on the relation ≿ which, when met, entail the existence of an interval-scale representation of the difference structure. That is, they entail the existence of a real-valued function Ψ defined on *A* that is unique up to a positive linear transformation, such that the biconditional (3) holds: (Krantz et al., 1971):


(3) ab≽cd if,and only if Ψ(a)−Ψ(b)≥Ψ(c)−Ψ(d)

The two main testable axioms of difference structures in the standard axiomatization (Krantz et al., 1971) are the weak ordering axiom, and the axiom of weak monotonicity or the sextuple condition. The *weak ordering axiom* requires that ≿ is a weak order (i.e., transitive and connected). It thus expresses the assumption, already discussed in Section 3.5, that people are able to consistently order intensity differences. The *sextuple axiom* is generally regarded as the central testable axiom of difference structures in the standard axiomatization (Krantz et al., 1971; Köbberling, 2006; see already Hölder, 1901). It is so called because it applies to sextuples of ordered stimuli *a* ≾ *b* ≾ *c* and *a*′ ≾ *b*′ ≾ *c*′, for which it requires the condition (4) to hold:


(4) Ifab≿a′b′andbc≿b′c′thenac≿a′c′

For the ~ part of ≿, axiom [4] reads: If *ab* ~ *a*′*b*′ and *bc* ~ *b*′*c*′, then *ac* ~ *a*′*c*′: If two adjoining intervals (judged intensity differences) *ab* and *bc* are equivalent in size to two other adjoining intervals *a*′*b*′ and *b*′*c*′, then the combined interval *ac* is equivalent to *a*′*c*′ (for a graphical illustration see Krantz et al., 1971, p. 145). The complete sextuple axiom merely extends this requirement by replacing ~ with ≿ (Krantz et al., 1971, p. 146). The sextuple axiom is an ordinal implication of the fact that intervals between numbers are additive: If two adjoining intervals on the number line, *x* – *y* and *y* – *z* are, respectively, identical to or greater than two other intervals *x*′– *y*′ and *y*′– *z*′, then the addition of the two intervals, *x* – *y* + *y* – *z* = *x* – *z*, is identical to (greater than) *x*′– *z*′. Additivity is the central condition that intensity intervals must meet, in addition to being weakly ordered, to allow an interval scale representation (Michell, 2012).

In alternative axiomatizations of difference structures, the sextuple axiom is replaced by a stronger requirement, the quadruple axiom (e.g., Debreu, 1958; Luce and Suppes, 1965; see also Köbberling, 2006), which requires: if *ab* ≿ *cd*, then *ac* ≿ *bd*. In our studies (Junge and Reisenzein, 2016), we tested this stronger axiom, partly to make up for the nontestability of the weak ordering axiom with GPCs (see Section 5.3). However, if the quadruple axiom is fulfilled, so is the sextuple axiom.

### 5.2 A realist and deductivist interpretation of RTM

The standard descriptions of RTM have been taken to imply by some authors (e.g., Borsboom, 2005) that RTM theorists interpret quantities *non-realistically* or *instrumentalistically*. That is, they regard the numerical representation of a qualitative structure (the scale Ψ) as an intervening variable that is useful as a compact summary of the ordinal relations in the data and as a device for making inferences, but does not refer to an independently existing quantity.

Furthermore, the standard descriptions of RTM suggest *a particular order of inquiry for the actual measurement process*. According to this order of inquiry, which can be called “inductivist” (and which is actually in tension with the otherwise deductive approach to measurement advocated by RTM theorists), the measurement process begins with the collection of a set of data for a qualitative relation structure, such as <*A* × *A*, ≿ > in the case of difference measurement. These data are next examined to determine whether they fulfill the axioms of the measurement structure. The actual measurement process, the estimation of scale values, is only performed in the third step (e.g., by applying a suitable nonmetric scaling method), and only if the second step has a positive outcome. This order of inquiry is nearly always followed in empirical applications of RTM (e.g., Schneider, 1982).

Although these interpretations of RTM undoubtedly reflect the views of some proponents of RTM, they are not shared by all (e.g., Orth, 1982; Westermann, 1983; Diéz Calzada, 2000). More importantly, the mathematical core of RTM—the qualitative relation structure, the representing numerical structure, the axioms, and the representation and uniqueness theorems derived from them—is equally compatible with a realist interpretation of quantities, and a deductivist approach to axiom testing.

#### 5.2.1 A realist interpretation of RTM

According to the realist view of quantities—that we endorse for at least some mental quantities including emotions—quantitative variables exist (or are hypothesized to exist) prior to and independent of any attempts to measure them, and the process of measurement is the attempt to determine the levels of the variable in a specific case (here and in part of what follows, we rely on Michell, 1999, 2005). As argued by Borsboom (2005), a realist view of quantities fits naturally with latent variable theories, to which ODS and MLDS belong.

As pointed out by Michell (1999), the concept of quantity (quantitative magnitude) was first defined in fully explicit and precise form by Hölder (1901, see Michell and Ernst, 1996, 1997) in his axioms of quantity. According to Hölder (1901), quantities are continuous variables whose levels are different degrees or gradings of a homogenous property, that stand to each other in a specific set of relations that together constitute an additive structure (Michell, 1999, 2005). Like the quantitative variable levels themselves, the relations between them may or may not be directly observable. In the latter case, which is characteristic for psychological quantities, what is observable—at least by the scientist—are only the manifestations or causal effects of the latent quantity in empirical measurements.

This realist view of latent quantities implies, among others, that the metric structure of the same latent variable (1) can manifest itself in somewhat different observable ways in the data resulting from different measurement procedures; (2) can get partly or completely lost in an attempted measurement process (e.g., O’Brien, 1985); and (3) that, as assumed in latent-variable measurement theories, measurements are always contaminated with some degree of error.

Furthermore, from a realist perspective, the assumptions (a) that a latent variable posited in a substantive theory (e.g., an emotion theory) is quantitative, and (b) that a particular measurement of this variable has a certain metric scale level (interval, ratio), are just two additional empirical assumptions made when testing the theory. The first assumption is implicitly made whenever a substantive theory postulates quantitative functional relations between variables, for these are only meaningful for quantitative variables. The second assumption is implicitly or explicitly made whenever researchers attempt to test the quantitative relations postulated in the theory by measuring their variables, for such tests are only meaningful if the measurements preserve (enough of) the variables’ metric structure.

Although the “metricity” assumptions [a] and [b] are structural rather than causal (see Michell, 1999), they can, in principle, be tested like other theoretical assumptions; that is, by deriving testable consequences from them and then testing these consequences. Generally speaking, metricity assumptions have two kinds of testable implications. First, the substantive theory **T**_**s**_, together with an associated measurement theory **T**_**m**_ (these are linked by their reference to the same quantities), entail that the quantitative relations among the latent variables postulated in **T**_**s**_, will also be observed for the measurements of these variables up to the scale level of the measurements, and up to measurement error. Therefore, one can test the metricity assumptions, if indirectly and holistically, by testing the empirical predictions of the theory with a set of measurements that one simultaneously hypothesizes to be metric. This is the classical approach taken in tests of latent-variable structural equation models (e.g., Kline, 2016), where the causal model and the measurement model are simultaneously estimated. Essentially the same holistic test of measurement assumptions is advocated in Anderson’s (Anderson, 1981, 1982) functional measurement method.

Second, **T**_**m**_ entails that the measurements of the latent variable fulfill, up to random error, the axioms of appropriate RTM measurement structures (see 5.2.2). This test of metricity is independent of **T**_**s**_ and therefore more diagnostic. However, analogous to the holistic test of metricity assumptions, a realist interpretation of latent quantities suggests a deductive rather than inductive order of inquiry when testing measurement axioms.

#### 5.2.2 A deductivist order of inquiry for testing measurement axioms

The deductivist order of inquiry in the measurement process has been elaborated in a series of papers by Westermann (1982, 1983, 1985). It begins with a proposed numerical measurement of a latent variable (e.g., scale values estimated by ODS) and only subsequently tests whether the scale values fulfill the axioms of an appropriate measurement structure (a closely related approach was proposed by Orth, 1982). In the context of the probabilistic difference scaling models, the deductive test of measurement axioms appears as just another diagnostic test, performed after the scaling, of the assumptions underlying the scaling model (see Maloney and Yang, 2003; Knoblauch and Maloney, 2008). A major benefit of testing measurement axioms in the context of probabilistic difference scaling models is that doing so provides a solution to a long-standing problem of RTM (see Krantz et al., 1971; Luce et al., 1990), the problem of accounting for measurement errors: Because ODS and MLDS are probabilistic latent variable models, they automatically yield an estimate of judgment error that can be used to construct a statistical test of axiom adherence (see Section 5.3).

Note, however, that the deductive order of inquiry for testing measurement axioms suggests an important modification regarding *how*, precisely, measurement axioms are tested (Junge and Reisenzein, 2016). Generally speaking, a measurement axiom is tested by selecting cases that fulfill the antecedent (if) condition of the axiom, and then checking whether these cases also fulfill the consequens (then) part of the axiom. In the classical RTM approach, this test, illustrated for the sextuple axiom, is implemented as follows: One selects sextuples of stimuli (*a*, *b*, *c*, *a*′, *b*′, *c*′) from *A* in <*A* × *A*, ≿ > that fulfill the condition *ab* ≿ *a*′*b*′ and *bc* ≿ *b*′*c*′, and then checks whether these sextuples also fulfill *ac* ≿ *a*′*c*′.

However, if the order of inquiry begins with actual (proposed) numerical measurements, it is only consequential, as well consistent with the general deductive approach to theory testing, to use the *estimated scale values* to select the antecedent cases of the axiom. The reason is that the scale values are the best available estimates of the latent variable values, and much less contaminated by error than is each individual comparative judgment (which is usually only made once). Hence, the deductivist approach suggests the following modification of the axiom test in ODS and MLDS (Junge and Reisenzein, 2016): The test cases are not chosen by relying on ≿ (for the sextuple axiom, by selecting sextuples of stimuli that fulfill the condition *ab* ≿ *a*′*b*′ and *bc* ≿ *b*′*c*′), but by selecting sextuples for which Ψ(*a*) – Ψ(*b*) ≥ Ψ(*a*′) – Ψ(*b*′) and Ψ(*b*) – Ψ(*c*) ≥ Ψ(*b*′) – Ψ(*c*′). For these sextuples, one then checks whether *ac* ≿ *a*′*c*′ is fulfilled in the empirical difference data (Junge and Reisenzein, 2016).

### 5.3 Testing the quadruple axiom

#### 5.3.1 The test procedure

As explained in Section 5.1, the two main testable axioms of difference structures are the weak ordering axiom and the sextuple axiom (or, in a different axiomatization, the stronger quadruple axiom). In our study on axiom adherence (Junge and Reisenzein, 2016) we could not test the weak ordering axiom, because this axiom is necessarily fulfilled if difference comparisons are derived from GPCs (see Orth, 1982; Junge and Reisenzein, 2016). However, as argued in Section 3.5, the assumption hat people can order differences of emotion intensity is intuitively plausible and there is evidence from difference scaling studies of sensations and perceptions that this axiom is usually fulfilled (up to random error). The focus of Junge and Reisenzein (2016) was therefore on the test of the quadruple axiom, which, as mentioned, implies the sextuple condition.

To test the quadruple axiom, we used a modified version of a parametric bootstrap test proposed by Maloney and Yang (2003) and Knoblauch and Maloney (2008) for testing axiom violation in the context of MLDS. This test was adapted to account for the fact that we used GPCs rather than QCs, meaning that the scale values and error variance were estimated by ODS rather than MLDS, and that the difference comparisons (*ab*; *cd*) were derived from the GPCs. Also different from Maloney and Yang (2003), we used a traditional performance criterion, the percentage of axiom adherence (= 100 – percent of axiom violations) as the test statistic. Most important, for reasons explained above, we used the estimated scale values instead of the participant’s ordinal judgments to select the test cases for the quadruple test.

Concretely, the axiom test was as follows. In the first step, the scale values estimated by ODS were used to select quadruples (*a*, *b*; *c*, *d*) that fulfilled the antecedent condition of the quadruple axiom. To account for the fact that participants cannot discriminate differences if they are too small, a conservative discriminability threshold was set. Furthermore, we selected only quadruples for which |Ψ_a_ – Ψ_b_| > |Ψ_c_ – Ψ_d_| (Orth, 1982) to account for the fact that small discriminable differences, that might still be detected in direct difference comparisons, cannot reveal themselves in GPCs because of the limited resolution of the response scale.

In the second step, the scale values and error variance of the judgments estimated by ODS were used to generate 10.000 simulated GPC responses, which were expanded to QCs. These simulated responses reflect the performance of an “ideal observer” (Maloney and Yang, 2003), i.e., a hypothetical twin of the participant who judges each quadruple according to the ODS model, given the participant’s scale values and error variance. From these simulated QCs, the ideal observer’s response to the antecedent of the quadruple axiom was extracted for the test cases of the axiom. Hence, the actual form of the tested axiom was: If |Ψ_a_ – Ψ_b_| > |Ψ_c_ – Ψ_d_| then *ac* ≻ *bd*.

In the third step, the percentage of correct responses to the test cases of the axiom (i.e., responses where *ac* ≻ *bd*) was computed for each simulation, and this performance index was accumulated into a bootstrap distribution. This distribution reflects the variability of the responses of the ideal observer who responds repeatedly to the axiom test cases. Finally, the percentage of correct responses of the participant was compared to the bootstrap distribution. If the probability of the obtained percentage correct was < 0.05, we concluded that the participant systematically violated the quadruple axiom. Otherwise, we concluded that the null hypothesis—the participant responded in accordance with the quadruple axiom—can be retained.

#### 5.3.2 Results

For the six emotions investigated by Junge and Reisenzein (2016), the hypothesis that the participants’ ODS scale values adhered to the quadruple axiom could be retained for most participants: amusement 71%; relief 74%; disgust 81%; surprise 88%; pleasantness 97%, and disappointment 97%. These findings suggest that the ODS scale values of most participants were metric or more precisely, interval-scaled. If one grants that the natural zero point of emotion intensity (the absence of emotion) was, with acceptable precision, estimated by the simultaneously collected direct intensity ratings, a ratio scale can be obtained for the axiom-conforming participants by linearly transforming their ODS values into the range of their intensity ratings (see Section 2.9).

### 5.4 Testing the metricity of direct scalings of emotion intensity

#### 5.4.1 The test procedure

If one accepts that the ODS scale of participants who passed the quadruple test is metric, one has a standard of comparison for deciding whether the direct emotion intensity scalings of these participants are metric as well. The underlying logic is this: If the emotion intensities estimated by ODS are interval-scaled, then any other interval-scale measurement *M* of the same emotion intensities is a linear transformation of the ODS scale and should therefore be linearly correlated with the ODS scale as highly as the reliability of the ODS scale and *M* permit. Based on this logic, Junge and Reisenzein (2016) constructed another bootstrap test to test the metricity of the direct emotion ratings. In this test, the ODS scalings were treated as error-free (which they nearly were), whereas the error contained in the ratings was estimated from the ratings’ re-test reliability (see Junge and Reisenzein, 2016).

For each participant and emotion, 10.000 simulated ratings were generated from the ODS scale by perturbing the scale values with normal error corresponding to that of the ratings. This procedure simulates a hypothetical twin of the participant who uses the ODS scale values to make the ratings, but makes random errors corresponding to the error level of the ratings. Each simulated set of ratings was then linearly correlated with the ODS scale values, and the correlations were accumulated into a bootstrap distribution. This distribution reflects the expected variability of the correlation between the direct and the ODS scale for a person who operates with the ODS scale values, but makes random errors in the ratings corresponding to the ratings’ error level. Finally, the bootstrap distribution was compared to the actual correlation between the direct and indirect scales obtained for the participant.

#### 5.4.2 Results

In Study 1 of Junge and Reisenzein (2016), 44% of the participants whose ODS scale values for pleasure were metric according to the quadruple test, and 23% of those whose ODS scale values of disgust were metric, also passed the metricity test for the corresponding ratings. Similar findings were obtained in Study 2 for ratings of amusement and surprise evoked by quiz items, and in Study 3 for ratings of disappointment about unobtained gains, and of relief about unobtained losses, in monetary lotteries. Hence, for all six investigated emotions, the direct ratings of emotion intensity of the majority of the participants deviated statistically significantly from the ODS scale values.

Notwithstanding the significant deviations from the metric (interval) scale level, it is reasonable to ask: Did the obtained direct ratings of emotion intensity at least *approximate* the linear ODS scale? A rough answer to this question is suggested by the size of the linear correlation between the direct and indirect scales of the participants who passed the quadruple test. In Study 1, this correlation was on average 0.80 for pleasure and 0.81 for disgust, although with a wide range (0.43 to 0.92 for pleasure and 0.18 to 0.96 for disgust). Similar correlations were obtained in Study 2 for surprise (*M* = 0.86, *range* = 0.67 to 0.94) and amusement (*M* = 0.88, *range* = 0.52 to 0.98) and in Study 3 for relief (*M* = 0.78, *range* = −0.18 to 0.96) and disappointment (*M* = 0.80, *range* = −0.36 to 0.96). Judged by traditional psychometric standards, the average obtained correlation of 0.82 would be considered fair. Thus, despite the statistically significant deviations of the emotion ratings of most participants from the interval scale level, the majority seemed to approximate linearity to a fair degree. This conclusion supports the assumption (e.g., Anderson, 1981, 1982) that the response function of carefully constructed rating scales is approximately linear. Although far from perfect (*R*^2^ = 0.67), the found degree of approximation of the ratings to the linear scale (represented by the ODS scale) may be sufficient for some kinds of analyses. However, as demonstrated by the results of Junge and Reisenzein (2013), emotion ratings are not precise enough and/or not close enough to metric to support tests of emotion theories on the individual subject level.

## 6 When can and should difference scaling be used?

Although we have focused on emotional experiences in this article, the proposed measurement approach can also be used to measure the intensity of sensations, bodily feelings, and other mental states characterized by an experiential quality of varying intensity. As mentioned, applications of difference scaling methods in both the older (e.g., Orth, 1982; Schneider, 1982) and more recent psychological literature (e.g., Boschman, 2001; Maloney and Yang, 2003; Maloney and Knoblauch, 2020) found that these methods yield precise measurements on an interval scale level for a variety of sensations and perceptions. Regarding the measurement of yet other mental states, particularly those whose conceptualization as quantities is a priori uncertain, caution is indicated (see Michell, 2012); in these cases, the proposed deductive method of testing measurement axioms could help to clarify the situation.

Despite the advantages of difference scaling methods, specifically ODS, for measuring the intensity of emotions, they are not the method of choice in all situations. This is so for two main reasons (see also, Junge and Reisenzein, 2013). First, like other indirect scaling methods, difference scaling cannot be used in all measurement contexts. In particular, it cannot be used when it is not possible or meaningful to compare multiple affective stimuli, or to present them repeatedly in GPCs or QCs. This is often the case in real-life situations (e.g., emotional reactions to outcome of exams; Pekrun and Bühner, 2014). Even in the laboratory, repeated stimulus comparisons are problematic for stimuli such as tastes and smells (Cardello, 2017).

Second, even when difference scaling methods are applicable, they are—again like other indirect measurement methods—more costly than direct scaling methods in terms of the time, effort and resources required for data collection and the calculation of scale values (Cardello and Jaeger, 2010; Cardello, 2017). However, it should be noted that these costs can be substantially reduced through computerized stimulus presentation, data collection, and scale value estimation (see Knoblauch and Maloney, 2008; Junge and Reisenzein, 2013). Although a time disadvantage in data collection remains, it is in fact not very large for ODS with up to about 12 stimuli, especially if the alternative consists of direct scalings repeated once (to increase reliability). For example, with 10 stimuli, there are 45 possible GPCs, but it appears that this number can be reduced by half without significantly degrading the scale value estimates (Boschman, 2001). This results in a comparable number of judgments to those needed for once-repeated, direct stimulus ratings. For 12 stimuli, the choice is between 24 ratings and about 30 GPCs. Furthermore, the time required to complete a GPC judgment is similar to that needed for a rating, and GPCs seem to be no more difficult to make than ratings. However, one potentially important difference remains: GPCs require twice as many stimulus presentations (2 in each trial) than direct scalings.

Whether the additional costs of difference scaling methods—even those of the economical ODS method—are an acceptable trade-off for obtaining more precise, less biased, and closely metric measurements, depends, among other factors, on the research question. Difference scaling methods are likely most useful in basic research when high-precision, metric measurements are desired to test substantive theories, particularly quantitative theories tested at the level of the individual. In contrast, in applied settings, where time constraints are often a preeminent concern, or when less precise and only roughly metric measurements are sufficient, difference scaling methods can be inefficient, i.e., too costly for the additional information they provide. In these situations, as well as in settings where difference scaling cannot be used (see above), optimized versions of the classical rating scale (see Anderson, 1982), or the newer labeled affective magnitude scales mentioned in Section 2.2.3, are currently (still) the best alternatives. And in some research contexts, ordinal or even qualitative (presence/absence) assessments of emotion will do.

Finally, even if the intensity of emotions is measured by ratings or other direct scaling methods, difference scalings are useful for checking the scale level obtained with these methods (Westermann, 1983).

## Data availability statement

The data analyzed in this study is subject to the following licenses/restrictions: no new data are reported. The datasets of our original, published studies referred to in the article will be made available to qualified researchers. Requests to access these datasets should be directed to rainer.reisenzein@uni-greifswald.de.

## Ethics statement

Ethical approval was not required for the study involving humans in accordance with the local legislation and institutional requirements. Written informed consent to participate in this study was not required from the participants or the participants’ legal guardians/next of kin in accordance with the national legislation and the institutional requirements.

## Author contributions

RR: Writing – original draft, Writing – review & editing. MJ: Writing – original draft, Writing – review & editing.
